# Composition Characterization of Fatty Acid Zinc Salts by Chromatographic and NMR Spectroscopic Analyses on Their Fatty Acid Methyl Esters

**DOI:** 10.1155/2019/7594767

**Published:** 2019-12-23

**Authors:** Kwang Seo Park, Yun Ju Kim, Eun Kyung Choe

**Affiliations:** Regulatory Chemical Analysis Laboratory, Korea Institute of Industrial Technology, Ansan 15588, Republic of Korea

## Abstract

To implement EU REACH- (Registration, Evaluation, and Authorization of Chemicals-) like chemical legislations in various countries of which the purpose is human and environment safety, the first step is substance identification followed by the hazard and risk assessments. Although both structural and composition identifications are required, the latter can more importantly result in the essential data to fill out the required substance information such as purity and concentrations of constituents, as well as impurities. With fatty acid zinc salts (FAZSs) as an exemplary industrial chemical of which chromatographic and nuclear magnetic resonance (NMR) analyses were impossible due to their insolubility in water and any organic solvents, the composition characterization was tried by preparing their fatty acid methyl esters (FAMEs) using the conc. HCl/methanol/toluene method. This acid-catalyzed methyl esterification was optimized with zinc stearate as a surrogate substance. Gas chromatography-mass spectrometry (GC-MS) and NMR analyses on methyl-esterified products revealed that the optimum conditions were at 90°C for 10 min or 45°C for 30 min with two equivalent HCl as well as at 45°C for 10 min with five equivalent HCl. Almost all zinc stearates were converted into the corresponding fatty acids with 97–99% conversion rates. Free fatty acids (FFAs) were detected in extracted ion chromatograms of pyrolysis-gas chromatography-mass spectrometry (Py-GC-MS) in the methyl-esterified products with incomplete conversions of 73∼79%. The optimized conc. HCl/methanol/toluene method of direct one-step reaction from FAZSs was compared with the two-step NaOH saponification/BF_3_-methanol method after acidic hydrolysis of FAZSs. The mechanism of fatty acid zinc salts into free fatty acids and fatty acid methyl esters was suggested with the evidence of the formation of Zn(OH)_2_.

## 1. Introduction

Substance identification is the cornerstone of the implementation of any chemical legislation. Accurate characterization of a substance is a prerequisite to various regulatory processes, especially in REACH [1]. The identity and concentration of the constituents of the registered substance as well as any impurities and additives should be reported. Compositional differences may affect the hazard and risk assessment of the substance and its classification [2, 3].

Fatty acid zinc salts (FAZS) are industrially produced by the reaction of fatty acids and ZnO [4] and have wide applications as components of lubricants, thermal stabilizers, processing additives in the natural and synthetic rubber production, and in the tire production with annual tonnages of 100–1000 or 10,000, for example, in Europe [5–8]. Fatty acids belong to the oleochemical substances that are derivatives from animal and vegetable oils. Crude glycerine and crude mixed fatty acids with different carbon chain length distribution and the degree of saturation are produced by hydrolysis depending on the initial fat or oil [9, 10]. Due to the variability in the composition of the starting materials, many oleochemicals become UVCB (Unknown or Variable composition, Complex reaction products or Biological materials) substance according to the definition of REACH regulation [2, 3, 10]. When FAZS need to be registered in the respective country according to its chemical legislation, its variable composition characterization should be followed.

After awareness of the suspected role that fats played in adverse health effects such as heart disease, rapid methods for analyzing fats and fatty acids were urgently needed in the middle of 1990's. Well-established methods for converting lipids including fatty acid, triglycerides, phospholipids, and sterols to fatty acid methyl esters (FAMEs) have been developed to analyse their total fatty acid components by gas chromatography ever since [11–18]. As reviewed shortly in Table 1, typical convenient and useful methods for converting various lipids into FAMEs include the acid-catalyzed esterification/transesterification and alkali-catalyzed transesterification, as well as boron trifluoride-methanol method. Methods A, B, and C can convert triglycerides and sterols into FAMEs while free fatty acids (FFAs) are not converted into methyl esters by the method C, alkali-catalyzed esterification. The BF_3_-methanol method belongs also to the acid-catalyzed reaction [11] while presaponification or pre-HCl hydrolysis of lipids facilitates the transesterification [14–17]. Thus, a rapid saponification followed by esterification with BF3-methanol in the same vessel as well as HCl hydrolysis followed by esterification with methanolic BF_3_ became national test standards in analyzing food lipids [15, 17].

In contrast to these many researches on lipid pretreatment methodology in an area of high general interest such as foods or human blood, esterification of these industrial chemicals like fatty acid metal salts was not reported elsewhere. In this study, proper methods for methyl esterification of FAZS were explored with zinc stearate as a surrogate substance. The total fatty acid compositions of FAZS of two industrial chemical products will be determined by GC-MS analysis on their FAMEs prepared by the optimized methods. Structural and purity confirmation on the FAMEs will be followed by NMR and Py-GC-MS analysis.

## 2. Materials and Methods

### 2.1. Chemicals

Hydrochloric acid (35.0%, Samchun Chemical, Korea), methanol (anhydrous, 99.9%, Samchun Chemical, Korea), and triundecanoin (98%, Sigma-Aldrich) were used. Boron trifluoride-methanol solution (14% in methanol) of A.C.S reagent grade and organic solvents (isooctane, ethyl acetate, and anhydrous toluene) of the GC analysis grade were purchased from J. T. Baker (PA, USA). Sodium chloride (99.5%, Sigma-Aldrich), sodium hydroxide (99%, Samchun Chemical, Korea), anhydrous sodium sulfate (Kanto Chemical Co., Japan), and magnesium sulfate (99.0%, Daejung Chemical, Korea) were used. Standard mixture of 37 fatty acid methyl esters (18919-1AMP Supelco FAME mix, certified reference material) was purchased from Sigma-Aldrich (St. Louis, MO, USA), and concentrations of each analyte were in the range of 2 wt.%∼6 wt.%. Distilled and deionized water of 18.2 MΩ was used.

### 2.2. FAZS Samples

Two kind samples of fatty acid zinc salts were provided from the manufacturer in Korea with the information of their origin. One sample (sample name: industrial chemical A) was manufactured from tallow and another sample (sample name: industrial chemical B) from plants. Commercial zinc stearate was used as a surrogate substance to explore the method for preparing methyl esters of fatty acids zinc salts and to optimize the esterification reaction. Zinc stearate (purum, Zn 11%, batch number BCBV6941) from Sigma-Aldrich was used as received.

### 2.3. Preparation of FAMEs

#### 2.3.1. Methyl Esterification of Zinc Stearate by the Conc. HCl/Methanol/Toluene Method

To 1 g of zinc stearate in a flask, 10 g of anhydrous toluene and 20 g of anhydrous methanol were added. After dropwise addition of the mixed solution containing 2 g of conc. HCl and 5 g anhydrous methanol to the flask, the resulting solution was heated with stirring at 90°C for 10 min, 30 min, or 60 min, respectively. The reacted solution was cooled to RT, and all the solvent was evaporated using a rotary evaporator. A portion of 30 mL of ethyl acetate was added to the crude product and washed with 50 mL of saturated NaCl solution three times. The separated ethyl acetate portion was dehydrated with MgSO_4_, evaporated, and dried using a vacuum pump. Reaction temperature of room temperature, 45°C or 90°C as well as the HCl amount of 1, 2, or 5 molar equivalents to that of zinc stearate were investigated to optimize the esterification reaction. The weight of the dried product was measured to calculate the yield of each reaction.

#### 2.3.2. Methyl Esterification of FAZS by the Conc. HCl/Methanol/Toluene Method

To 1 g of fatty acid zinc salts (industrial chemical A and B, respectively) in a flask, 10 g of anhydrous toluene and 20 g of anhydrous methanol were added and heated to 90°C. The same procedure in Section 2.3.1 was carried out with 2 equivalents of HCl and reaction time of 10 min.

#### 2.3.3. Methyl Esterification of FAZS from Hydrolysed Samples

*(1) Hydrolysed Samples*. One gram of FAZS (industrial chemical A and B, respectively) was heated with 25 mL of distilled water and 5 mL of hydrochloric acid at 150°C for 2 h. After cooling to room temperature, the hydrolysed sample floating as a solid (industrial product A) or oil layer (industrial product B) was separated from the aqueous layer by filtration and subjected to the below reaction that is a well-established method for fatty acids to FAMEs.

*(2) NaOH Saponification and Methanolic BF_*3*_ [15]*. To 15 mg of the hydrolysed sample prepared in (1), 1 mL of the internal standard solution (triundecanoin 1000 mg/L) and 1.5 mL of 0.5 N NaOH in methanol were added and heated at 80°C for 5 min. A portion of two mL of 14% BF_3_ in methanol was added and heated at 80°C for 30 min. Then, 1 mL of iso-octane was added and shaken for 30 sec. Five mL of saturated aqueous NaCl solution was added immediately, covered, and shaken. The iso-octane layer was separated and dried with anhydrous sodium sulfate.

### 2.4. Measurements

#### 2.4.1. NMR Measurements

The NMR sample solutions for hydrolysed samples in (1) were prepared by dissolving 5 mg of each sample in DMSO-d_6_ (0.5 mL). For complete dissolution of the FAME sample from industrial chemical B, additional CDCl_3_ (0.1 mL) as well as DMSO-d_6_ (0.5 mL) were used. Those for FAMEs from zinc stearate and industrial chemical A were prepared in CDCl_3_. The proton NMR measurements were performed using a Bruker Avance III 400 MHz NMR spectrometer.

#### 2.4.2. GC-MS Measurements

The sample solution was prepared by dissolving 20 mg of the methyl-esterified sample (FAMEs) in methylene chloride (2 mL). A 1 *μ*l aliquot of iso-octane solution was injected in an Agilent 7890B GC with a 5977A quadrupole mass spectrometer. Separation was carried out with a SP-2560 capillary column (*L*: 100 m, I.D.: 0.25 mm, *d*_f_: 0.2 *μ*m, biscyanopropyl phase). Injector and detector temperatures were 225°C and 230°C, respectively. The split injection mode was used with a split ratio of 200 : 1. Helium was used as the carrier gas at a flow rate of 0.75 mL/min. The temperature ramp program was as follows: the initial column temperature was 100°C, which was kept for 4 min, and then the temperature was raised to 240°C at 3°C/min, where it was maintained for 15 min. The mass scan range was 29∼600 m/z. The mass detector was operated in the electron ionization mode with cone voltage of 70 eV. Individual FAME was identified by comparing its retention time and the corresponding mass spectrum with those of known FAMEs that were prepared by dissolving 100 mg of standard mixture `containing 37 FAMEs in 4 mL methylene chloride ([Supplementary-material supplementary-material-1]).

#### 2.4.3. Py-GC-MS Measurements

To check whether FFAs were formed as by-products during the esterification reactions, Py-GC-MS analyses were carried out. A temperature-programmable pyrolyzer (PY-2020iD, Frontier Laboratories, Ltd., Japan) was coupled with GC-MS (7890/5975, Agilent Technologies, USA). Esterified reaction product (0.5 mg) by 2.4.1 was loaded as a solid into the stainless steel cup (80 *µ*L Disposable eco cup, Frontier Lab). Then, the sample cup was placed in a furnace at 600°C for 1 min. The split injection mode was used with a split ratio of 100 : 1. The thermally desorbed components were separated with a HP-5MS UI column (*L*: 30 m, I.D.: 0.25 mm, *d*_f_: 0.25 *μ*m, (5%-Phenyl)-methylpolysiloxane). The temperature of the column was kept at 50°C for 1 min, then raised to 320°C at 10°C/min, and maintained for 20 min. Interface temperature between the furnace in the pyrolyzer and the GC was 320°C. Helium was used as the carrier gas at a flow rate of 1.0 mL/min. The mass temperature was 300°C, and the mass scan range was 29∼800 m/z. After confirming the retention times of FFAs by total ion chromatograms, extractable ion chromatograms were obtained using m/z 256 and m/z 284 for C16 : 0 and C18 : 0 fatty acids, respectively.

### 2.5. Calculations of Conversion and Total Fatty Acid Composition

Conversions of FAZSs to the corresponding methyl esters after the reaction in Section 2.3.2 were estimated both by NMR peaks as well as GC-mass chromatograms. Conversion using ^1^H NMR spectra was calculated as the ratio of A to A plus B where A is an integrated area of C2 protons of fatty acid (methylene protons *α* to the carboxylic acid group) and B is that of C2 protons of FAMEs (methylene protons *α* to the methyl ester group). Total fatty acid composition in FAZSs were calculated by converting the FAMEs measured by GC-MS to the corresponding fatty acids. An example showing the calculation from peak areas of FAMEs from the sample and internal standard to the total fatty acid composition is presented in [Supplementary-material supplementary-material-1]. Without internal standard, relative peak areas of each FAME were used for comparison of total fatty acid composition.

## 3. Results and Discussion

### 3.1. Optimization of Methyl Esterification of Zinc Stearate by the Conc. HCl/Methanol/Toluene Method

Zinc stearate was used as a surrogate substance of FAZSs. It was found to consist of C16 : 0 and C18 : 0 FAZS with other minor components of saturated fatty acid zinc salts as would be discussed in Section 3.1.3.

#### 3.1.1. Esterification Conditions with Three Parameters

Zinc stearate was not soluble in conc. HCl-methanol solution alone; toluene was added as an inert solvent to effect a reaction solution like FAME preparations from various lipids previously reported [11–13, 16, 18]. Zinc stearate did not dissolve in toluene. However, dropwise additions of conc. HCl-methanol solution made a clear solution gradually. Raising temperature from room temperature (RT) to 45°C or 90°C allowed the reaction to proceed faster. Toluene helped zinc stearate to turn into the corresponding methyl esters by making zinc stearate partly soluble in a raised temperature as well as making the reaction product soluble. Becoming a clear solution was a signal that the reaction of methyl esterification proceeded and was completed.  Table 2 showed measured data for yields and conversion of zinc stearate to stearic acid methyl ester under 12 different reaction conditions of this conc. HCl/methanol/toluene method. Three reaction parameters including temperature, time, and amount of HCl per zinc stearate were varied to optimize the acid-catalyzed esterification reaction of zinc stearate [19], while addition of toluene was regarded as an essential parameter. NMR and GC-MS measurements on methyl-esterified products from zinc stearate were used to evaluate each reaction. Because methyl-esterified products were soluble in chloroform or methylene chloride, NMR spectra were taken using deuterated chloroform (CDCl_3_) and samples were dissolved in methylene chloride for GC-MS analyses.

#### 3.1.2. Monitoring of Methyl Esterification of Zinc Stearate by NMR

**Figure 1:**
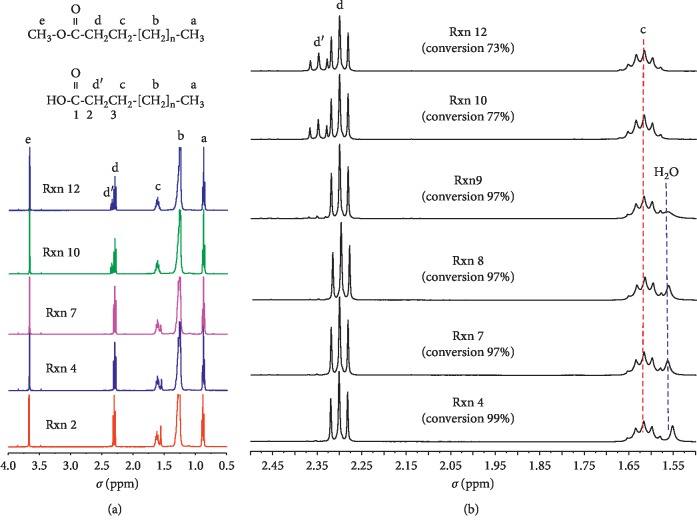
Comparison of NMR spectra using CDCl_3_ of methyl-esterified products obtained from zinc stearate in various reaction conditions: a (0.86 ppm), b (1.17–1.36 ppm), c (1.62 ppm), d (2.30 ppm), d′ (2.35 ppm), and e (3.67 ppm).

Five different proton peaks were observed in NMR spectra (Figure 1-a) of esterified products from reactions 2, 4, and 7 having relatively superior reaction conditions in terms of time, temperature, and equivalent amount of HCl (mole of HCl per mole of zinc stearate). From the upfield, protons of the terminal methyl (peak a, 0.86 ppm, triplet, 3H), methylene protons of stearyl and palmityl (peak b, 1.17–1.36 ppm, multiplet, 26.05H), methylene protons of C3 (peak c, 1.62 ppm, double triplet, 2.03H), methylene protons of C2 (peak d, 2.30 ppm, triplet, 1.99H), and methoxy protons (peak e, 3.67 ppm, singlet, 2.83H) were measured in the NMR spectra ([Supplementary-material supplementary-material-1]). In addition to these peaks, another triplet peak was detected at 2.35 ppm (peak d′) in the products from relatively poor conditions such as reactions 10 and 12. This peak can be reasonably assigned to be methylene protons of C2 of free stearic and palmitic acids that were produced as by-products. While peaks a, b, and c of FAMEs and FFAs occurred at the same field, the methylene protons of C2 absorptions of FFA occurred at the slightly downfield compared with methyl stearate as shown in Figure 1-a (peak d′ vs. peak d). This shift can be explained by the environment of the protons of FFA in *α* position to more electronegative carboxylic acid compared with those in *α* position to methyl ester.

Conversion of FAZSs to FAMEs in each reaction could be calculated using the integration area at 2.30 ppm (peak d) and 2.35 ppm (peak d′) as mentioned in Section 2.5. Peak assignments in Figure 1 were consistent with the previous reports in the area of food research [20–22]. The peak around 1.56 ppm could be attributable to water. It is well-known that a proton peak of H_2_O in D_2_O is commonly observed around 4.8 ppm due to hydrogen bonding while a proton peak of H_2_O in DMSO-d6 and CDCl_3_ shift to the upfield such as 3.33 ppm and 1.5∼1.6 ppm, respectively [23–25].

#### 3.1.3. Monitoring of Methyl Esterification of Zinc Stearate by GC-MS

**Figure 2:**
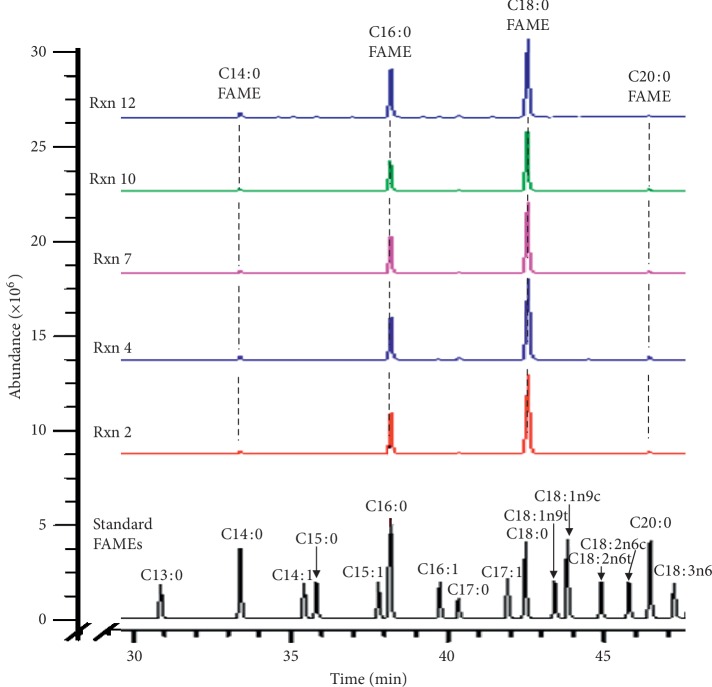
GC-mass chromatograms of methyl-esterified products (FAMEs) from zinc stearate.

**Figure 3:**
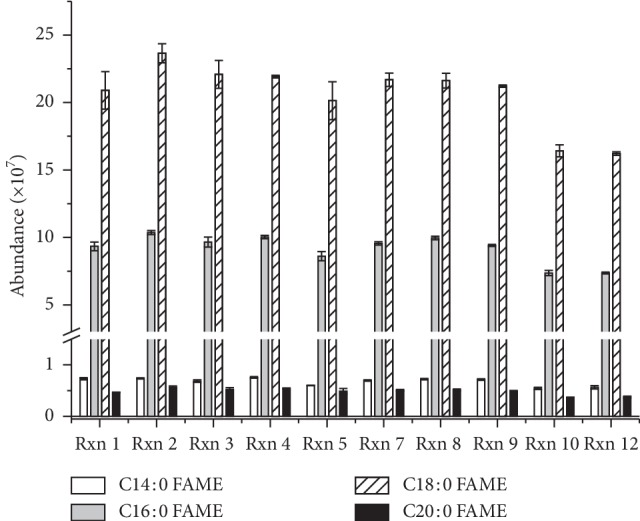
Compositions of fatty acids in methyl-esterified products obtained directly from zinc stearate in various conditions. The areas of each peak were plotted.

GC-MS analysis on methyl-esterified products dissolved in CH_2_Cl_2_ revealed that zinc stearate consisted of C16 : 0 and C18 : 0 fatty acid zinc salts as major components as well as C14 : 0 and C20 : 0 fatty acids zinc salts and trace amounts of other C12 : 0, C15 : 0, C17 : 0, and C20 : 0 fatty acid zinc salts (Figure 2). C16-FAME and C18-FAME in methyl-esterified products were confirmed by comparing their retention times with those of the standard mixture of FAMEs (38.1 min and 42.5 min, respectively) ([Supplementary-material supplementary-material-1]). Relative amounts of the C14, C16, C18, and C20 fatty acid chain in zinc stearate substance seemed to be maintained after esterification under the different conditions (Figure 3). The ratio of peak area between C16 and C18 FAZS was estimated to be approximately 1 : 2.2∼2.3 in all twelve reactions. Our result supported the previous finding that all fatty acids are known to be esterified at approximately the same rate by methanolic hydrogen chloride, so there are unlikely to be differential losses of specific fatty acids during the esterification step [11].

#### 3.1.4. The Conc. HCl/Methanol/Toluene Method Optimized

Among twelve different conditions of the conc. HCl/methanol/toluene method, conversion of zinc stearate to the corresponding FAME was found to be more than 97% only if more than two equivalent mole of HCl was used per mole of zinc stearate. However, when one or half equivalent mole of HCl was used, esterification proceeded incompletely regardless of a high reaction temperature of 90°C (reaction 4 and 5). With two equivalent amount of HCl, esterification at 90°C for 10 min resulted in 99% conversion (reaction 4), while reaction time for 30 min was required at 45°C to give 97% conversion (reaction 7). Esterification at 45°C with five equivalent moles of HCl need esterification time only for 10 min to give the same conversion (reaction 9). At room temperature, however, esterification for 30 min even with five equivalent moles of HCl resulted in incomplete conversion (73% in reaction 12). Higher reaction temperature and longer reaction time, as well as larger amount of HCl resulted in the complete conversion of zinc stearate and zinc palmitate to methyl stearate and methyl palmitate. Under the most extreme condition of 90°C for 1 h with five equivalent amount of HCl, the conversion was calculated to be 99% and there seemed to be no side reaction product. Reactions 4, 8, and 9 could be suggested as the optimum conditions for methyl esterification of zinc stearate with conversion of 97%∼99%. Approximately 5% of weight loss was observed by measurement of weights of zinc stearate as the starting substance and final product after correcting the weight of Zn. The weight loss was possibly during the work-up process such as evaporation of toluene, extraction with ethyl acetate, cleaning-up with water three times, and dehydration and filtering, resulting in an average yield of 95% of the esterification product.

#### 3.1.5. Identification of By-Products

In the condition of reactions 10 and 12, zinc stearates were not all converted to FAMEs, ending up with considerable amounts of FFAs. Formation of FFAs were evidenced by a new appearance of NMR peak at 2.35 ppm in CDCl_3_ attributable to CH_2_ of C2 in free stearic and palmitic acids (Figure 1). Conversion of zinc stearates to FAMEs was 77% and 73%, respectively as determined by NMR analysis while FFAs as by-product were estimated to be 23% and 27% of total products (Figure 1-b). This result agreed well with the conversion rates measured by GC-MS analysis which showed 79% conversion of zinc stearates with chain length C16 : 0 and C18 : 0 to their methyl esters (Table 2 and Figure 3).

**Figure 4:**
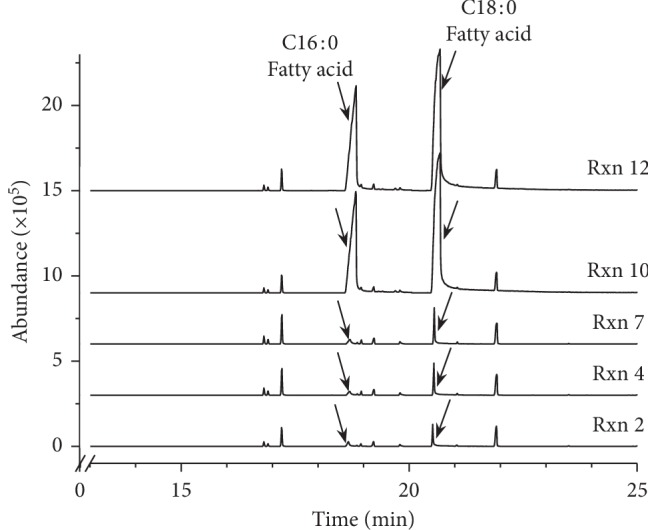
Results of Py-GC-MS analysis directly on methyl-esterified products (FAMEs) showing the free C16 : 0 and C18 : 0 fatty acids in Reaction 10 and Reaction 12. Extractable ion chromatogram was obtained using m/z 256 for C16 : 0 fatty acid in the range of retention time 10∼20 min and m/z 284 for C18 : 0 fatty acid in the range of retention time 20∼25 min.

FAAs were not detected during the GC-MS measurement of FAMEs using a highly polar SP-2560 capillary column in the range of retention time 0∼50 min. Therefore, Py-GC-MS analyses were carried out directly on methyl-esterified products without sample preparation conveniently. Exposure at 600°C for 1 min enabled FFAs to vaporize and be injected into the GC column. Free C16 : 0 and C18 : 0 fatty acids were successfully detected by the Py-GC-MS system using a low polar HP-5MS UI column at 18.8 min for C16 : 0 fatty acid and at 20.6 min for C18 : 0 fatty acid (Figure 4). Meanwhile, only trace amount of FFAs were produced in the reactions 2, 4, and 7 with conversion rates more than 97%, and relatively much more FFAs were found to be present as by-products in reactions 10 and 12 with conversion rates of 73–77% (Figure 4).

To avoid the formation of FFAs during the esterification, anhydrous HCl/methanol might be used. However, the amount of FFA formed due to the presence of water that was derived by using conc. HCl was less than 1% or 3% using the optimized reaction conditions (Table 2). A convenient method using commercial aqueous conc. HCl rather than anhydrous HCl/methanol was also recommended in preparation of FAMEs from fatty acids, triglyceride, phospholipids, and sterol ester as well as blood glycerolipids by Ichihara et al. [12].

### 3.2. Compositional Characterization of FAZS

The corresponding FAMEs of FAZSs were prepared in two different ways. First, FAZS A and B were esterified by the conc. HCl/methanol/toluene method using one of the optimized conditions proposed in 3.1.4 (90°C, 10 min, two equivalent mole of HCl). Secondly, FFAs were obtained by acidic hydrolysis of FAZS and were esterified using the well-established KFDA method [15] for comparison. The first method was a one-step reaction to prepare FAMEs directly from FAZS, while the second method was two steps including hydrolysis of FAZS by aqueous conc. HCl solution followed by esterification of FFA using NaOH Sap/methanolic BF_3_ with an internal standard (triundecanoin).

#### 3.2.1. Identification of FAMEs Prepared from FAZS by NMR

**Figure 5:**
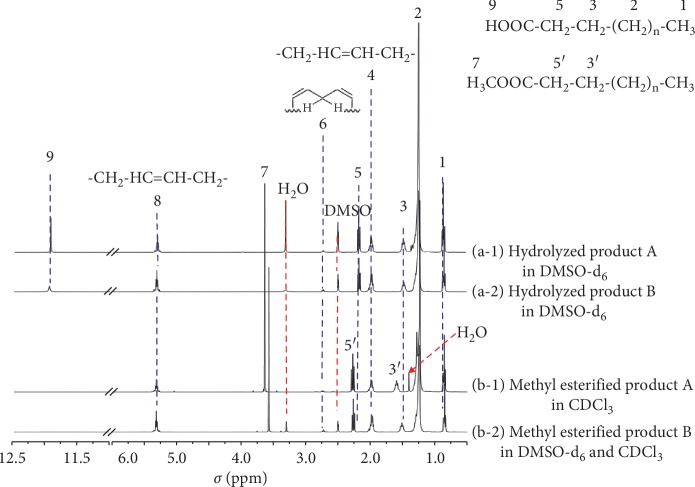
Stacked ^1^H NMR spectra of the hydrolysed products and the methyl-esterified products from two kinds of fatty acid zinc salts A and B.

For NMR measurements of FAMEs and the comparison with NMR spectra of FFA (Figure 5-a), DMSO-d_6_ of which ^1^H chemical shift of a residual proton occurs at 2.50 ppm [23] was tried to dissolve methyl-esterified products for the prevention of hydrogen bond effect on peak resonance. Methyl-esterified product B was soluble in DMSO-d_6_ with additional CDCl_3_ (ca. 5 : 1) while methyl-esterified product A was not readily soluble, so CDCl_3_ was used as its NMR solvent. The NMR peaks were consistent with those in FFA samples except that methoxy protons replaced protons of carboxylic acid group (Figure 5-b). NMR measurements proved that FAMEs were successfully prepared from industrial samples of FAZS like from the surrogate substance, Zn stearate. The peak 5′ (methylene protons of C2, triplet) of FAME samples appeared at 2.26–2.28 ppm in CDCl_3_ solution (Figure 5-b and [Supplementary-material supplementary-material-1]-b), showing a good agreement with the same peak in FAMEs prepared from zinc stearate at 2.30 ppm in CDCl_3_ (Figure 1).

Chemical shifts to downfield were observed with several peaks in CDCl_3_ when compared to the same peaks in DMSO-d6. While the peak 5 (methylene protons of C2) of FFA appeared at 2.17 ppm in DMSO-d6 solution (Figure 5-a and [Supplementary-material supplementary-material-1]-a), the peak d′ (methylene protons of C2) of FFA in CDCl_3_ occurred more downfield at 2.35 ppm (Figure 1). The peak 3′ (methylene protons of C3) and the peak 7 (methoxy protons in ester group) of FAMEs occurred at slightly downfield in CDCl_3_ (Figure 5-b-1 and [Supplementary-material supplementary-material-1]-b-1) than in DMSO-d6 plus CDCl3 (ca.5 : 1) (Figure 5-b-2 and [Supplementary-material supplementary-material-1]-b-2) by 0.08 ppm and 0.07 ppm, respectively. Interestingly, dependency of two different solvents on chemical shifts was not observed in other protons of carbons more than two adjacent to carbonyl group in carboxylic acid or methyl ester.

Hydrogen bonding leads to chemical shift downfield [23–25]. The solvent DMSO is known to make strong hydrogen bonds as a hydrogen acceptor so that it breaks up the intramolecular hydrogen bonds of dissolved molecules. Chloroform is a weak hydrogen acceptor not being able to disrupting intramolecular hydrogen bonds of dissolved molecules [26]. It has been reported that NMR peaks of protons involved in intramolecular hydrogen bonds appeared more downfield in CDCl_3_ than in DMSO-d_6_ due to intramolecular hydrogen bonds maintained in CDCl_3_ [27, 28]. Our observation of chemical shifts to downfield with protons in *α*- and *β*-positions seems to be related to possible intramolecular hydrogen bonds between oxygen in the carbonyl group and protons in *α*- and *β*-positions.

#### 3.2.2. Total Fatty Acid Compositions of FAMEs

**Figure 6:**
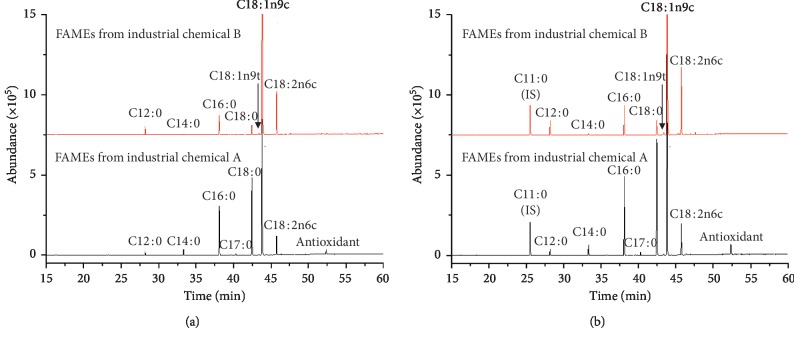
GC-mass chromatograms of methyl-esterified products prepared (a) directly from fatty acids zinc salts (industrial chemical A and B) by the conc. HCl/methanol/toluene and (b) by NaOH saponification/methanolic BF_3_ method on hydrolysed samples. The internal standard (triundecanoin) was methyl-esterified together.

GC-MS analyses on the FAMEs showed that FAZSs with C18 : 1(oleic, 47%), C18 : 0 (stearic, 28%), C16 : 0 (palmitic, 15%), and C18 : 2(linoleic, 7%) chains were main components in industrial chemical A, while FATZ with C18 : 1(oleic, 74%), C18 : 2(linoleic, 11.8%), C16 : 0 (palmitic, 5%), and C18 : 0 (stearic, 3%) chains constituted industrial chemical B (Figure 6 and Table 3). These GC-MS results on the main compositions agreed well with the physical observation of industrial chemicals A and B. Pellets of FAZS B with more unsaturated and less stearyl chains were more brittle than those of FAZS A. Their hydrolysed samples A and B were solid and liquid, respectively. A peak detected at 52.4 min (Figure 6) was proved to be a phenol-type antioxidant additive in industrial chemical A [29].

The comparison between total fatty acid compositions in each FAZS that were prepared in two different ways showed a good level of consistence (Table 3). Esterification of FAZS using the conc. HCl/methanol/toluene proved to be a fast convenient one-step method for preparing their FAMEs for compositional characterization.

### 3.3. Mechanism of Conversion of FAZS to FFAs or FAMEs

**Figure 7:**
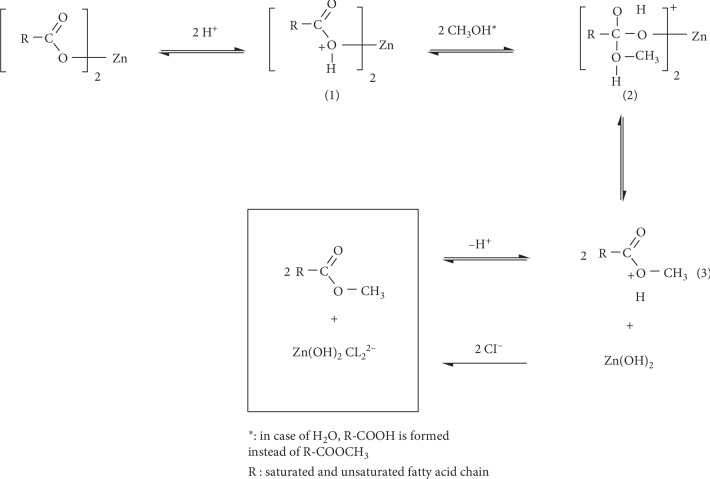
Proposed mechanism for formation of fatty acid methyl esters from fatty acid zinc salts.

Acid-catalyzed transesterification of lipids such as triglyceride is known to give an oxonium ion like (1) in Figure 7 by initial protonation [11]. Then, the oxonium ion undergoes an addition of alcohol to form an intermediate like (2). Dissociation of the intermediate via the transition state like (3) is followed to give the final ester. Based upon this general mechanism and our finding of zinc hydroxide in the final reaction solution ([Supplementary-material supplementary-material-1]), the mechanism for formation of FAME or FFA from FAZS was proposed in Figure 7. The mechanism can explain why at least two molar equivalents of HCl per FAZS were required for the fast complete conversion to FAMEs.

To support the proposed mechanism, the presence of Zn(OH)_2_ in our reaction medium was confirmed by observing changes in aqueous work-up layers of the reaction mediums by adding increasing amounts of NaOH(aq). The initial aqueous layer was a clear acid solution of pH 1 ([Supplementary-material supplementary-material-1]-a). However, it became turbid by adding a small amount of NaOH (pH 6.8) ([Supplementary-material supplementary-material-1]-b). White precipitation was observed by adding more NaOH (pH 13.2) ([Supplementary-material supplementary-material-1]-c). The solution became clear again by adding excess NaOH ([Supplementary-material supplementary-material-1]-d). It was well-known that zinc hydroxide is not soluble in water and in methanol. However, Zn(OH)_2_ forms a colorless, water-soluble complex Zn(OH)_2_Cl_2_^2^ with chloride ions and forms Zn(OH)_4_^2−^ in the excess of hydroxide ions [30]. Therefore, it seemed that Zn(OH)_2_ existed as the water-soluble form, Zn(OH)_2_Cl_2_^2^, in the initial acidic solution.

FAZSs as an exemplary industrial chemical of which chromatographic and NMR analyses were impossible due to their insolubility in water and any organic solvents. Compositions of FAZSs can be determined due to successful preparations of their FAMEs using the conc. HCl/methanol/toluene method. Composition characterization developed in this study can be applied to substance identification of chemical legislations worldwide as well as quality controls of FAZS chemicals for various industrial uses.

## 4. Conclusions

FAZSs were converted into their corresponding methyl esters (FAMEs) by a convenient one-step esterification reaction using the conc. HCl/methanol/toluene method. The optimized conditions were at 90°C for 10 min or 45°C for 30 min with a 2 : 1 ratio between HCl and FAZS, as well as at 45°C for 10 min with a 5 : 1 ratio between them. Compositions of industrial chemicals like FAZSs were successfully determined by GC-MS analyses on their FAMEs. Esterified reaction products were successfully evaluated by NMR and GC-MS measurements, giving good agreements between two measurements. FFAs were detected as by-products in not-optimized esterification reactions as measured by NMR and Py-GC-MS.

## Figures and Tables

**Table 1:** Simplified comparison of typical preparation methods for fatty acid methyl esters from various lipids.

Methods/reagent	Optimized or recommended conditions^a^	Analytes^b^	Ref./year
*(A) Acid-catalyzed esterification/transesterification*			
Anhydrous methanolic hydrogen chloride	Reflux, 2 h or 50°C, overnight (5% HCl)	Other than FFA	[11] 1993
	50°C, 30 min (5% HCl)	FFA	
Conc. HCl/methanol/toluene	45°C, 8 h (1.2% HCl)^c^	TG, PhL	[12] 2010
	45°C, 14 h (0.6% HCl)	TG, PhL	
	45°C, 16 h (1.2% HCl)	SE	
	45°C, 20 min (0.5% HCl)	FFA	
	45°C, 60 min (0.2% HCl)	FFA	
	100°C, 30 min (1.2% HCl)	TG, PhL	
	100°C, 90 min (1.2% HCl)	SE	
	100°C, 5 min (0.2% HCl)	FFA	

*(B) Esterification/transesterification by BF*_*3*_ *-methanol or modified BF*_*3*_ *-methanol*			
BF_3_-methanol	100°C, 2 min (14% BF_3_)	FFA	[13] 1964
BF_3_-methanol/onert solvent	100°C, 30 min (25% BF_3_)^d^ 100°C, 45 min (35% BF_3_)^d^	TG SE	
NaOH saponification/methanolic BF_3_	100°C, 5 min (0.5 N NaOH)/100°C, 2 min (12.5% BF_3_)	Cottonseed oil	[14] 1966
	100°C, 5 min (0.5 N NaOH)/100°C, 30 min (14% BF_3_)	Lipids in foods	[15] 2017 KFDA
KOH saponification/acidification/methanolic BF_3_	70°C, 1 h (0.5 M KOH)/2 M HCl/RT, 20 min (15% BF_3_)	Lipid mixture	[16] 1996
Hydrolysis by HCl/methanolic BF_3_	80°C, 40 min (8.3 M HCl)/100°C, 45 min (7% BF_3_)	TG, FFA in foods	[17] 2002 AOAC

*C. Alkali-catalyzed transesterification*			
Anhydrous sodium or potassium methoxide	50°C, 10 min (0.5 to 2 M CH_3_ONa, 100-fold excess)	TG	[11] 1993
	50°C, 5 min	PhL	
	50°C, 1 h	SE	
Methanolic NaOH or KOH	RT, 2 min (2 M KOH)^e^	TG	[16] 1996
	37°C, 1 min (2 M KOH)^e^	TG	
	RT, 15 h (0.1 M KOH)^e^	TG	
	37°C, 1 h (0.5 M NaOH)^c,f^ 37°C, 40 min (0.5 M NaOH)^c,f^	SE wax	[18] 2003

^a^Yield of FAME: 97%∼100%. ^b^Analytes are lipids: TG, triglyceride; FFA, free fatty acid; SE, sterol ester, cholesteryl ester; PhL, phospholipids. ^c^Concentration in final volume. ^d, e, f^Inert solvent is added: d, benzene; e, hexane; f, methyl propionate.

**Table 2:** Optimized conditions for preparation of FAMEs from zinc stearate^a^ by acid-catalyzed esterification using the conc. HCl/methanol/toluene method.

Rxn no.	Temp (°C)	Time (min)	Amount of conc. HCl	Yield^c^ (%)	Conversion (%)		
					By NMR	By GC-MS^e^	
						C16^e^	C18^e^
1	90	60	2 g (5 eq^b^)	94.1	>99	100.0 ± 3.4	100.0 ± 6.6
2	90	30	2 g (5 eq)	93.8	>99	110.9 ± 1.4	113.1 ± 3.0
3	90	10	2 g (5 eq)	94.6	>99	103.4 ± 3.8	105.7 ± 4.7
4	90	10	0.8 g (2 eq)	96.3	>99	107.3 ± 1.2	104.9 ± 0.5
5	90	10	0.4 g (1 eq)	94.9	91	92.2 ± 3.9	96.3 ± 7.0
6	90	60	0.2 g (0.5 eq)	—	Insoluble^d^	—	—
7	45	30	2 g (5 eq)	93.4	>97	102.3 ± 1.3	103.8 ± 2.3
8	45	30	0.8 g (2 eq)	96.1	>97	106.6 ± 1.3	103.4 ± 2.5
9	45	10	2 g (5 eq)	91.7	>97	100.7 ± 0.8	101.5 ± 0.4
10	45	30	0.4 g (1 eq)	94.4	77	78.8 ± 2.6	78.5 ± 2.7
11	45	30	0.2 g (0.5 eq)	—	Insoluble^d^	—	—
12	rt	30	2 g (5 eq)	91.9%	73%	78.8 ± 0.9	77.6 ± 0.8

^a,e^Zinc stearate consisted of C16 and C18 fatty acid zinc salts as major components and small amounts (less than 3%) of C14 and C20 fatty acid zinc salts. ^b^Equivalent number was calculated as mole of HCl per mole of zinc stearate. ^c^Yield was calculated based on weight measurements. ^d^Reaction mixture remained insoluble throughout the reaction. ^e^GC-MS data were expressed as means ± standard deviation (*n* = 3).

**Table 3:** Compositional characterization of two kinds of fatty acids zinc salts A and B by GC-MS analysis on their FAMEs prepared by the optimized conc. HCl/methanol/toluene method.

Peak no.	RT (min)	Fatty acids^a^	Total fatty acid composition (%)			
			Industrial chemical A		Industrial chemical B	
			Conc. HCl/MeOH/toluene^b^	NaOH sap/methanolic BF_3_^c^	Conc. HCl/MeOH/toluene^b^	NaOH sap/methanolic BF_3_^c^
1	28.18	C12 : 0	1.13 ± 0.18^d^	0.85 ± 0.03	2.35 ± 0.03	1.72 ± 0.04
2	33.34	C14 : 0	2.04 ± 0.22	1.62 ± 0.07	0.30 ± 0.01	0.12 ± 0.01
3	38.10	C16 : 0	15.46 ± 0.58	15.05 ± 0.60	5.00 ± 0.05	3.96 ± 0.06
4	40.30	C17 : 0	1.35 ± 0.01	0.86 ± 0.06	—	
5	42.46	C18 : 0	27.69 ± 0.45	27.44 ± 1.31	3.02 ± 0.02	2.22 ± 0.03
6	43.39	C18 : 1n9t	—	—	1.12 ± 0.01	0.49 ± 0.07
7	43.81	C18 : 1n9c	45.68 ± 0.49	49.15 ± 2.39	76.39 ± 0.08	77.82 ± 0.96
8	45.75	C18 : 2n6c	6.64 ± 0.05	6.36 ± 0.39	11.81 ± 0.03	10.79 ± 0.16
		Total	100.00	101.33	100.00	97.11

^a^Fatty acids are designated by number of carbons: number of doubles (C18 : 2n6c means C18 fatty acid with 2 adjacent cis double bonds starting at C6 counting from the methyl group). ^b^Esterified directly from fatty acids zinc salts. ^c^Esterified from the hydrolysed products (free fatty acids) of fatty acids zinc salts. ^d^GC-MS data expressed as means ± standard deviation (*n* = 3).

## Data Availability

No data were used to support this study.

## Supplementary Materials

Supplementary Materials The supplementary data consist of the following Tables S1–S3 and Picture S1: Table S1. Retention times and concentrations of 37 FAMEs in the standard solution. Table S2. Calculation of total fatty acids from GC-MS analysis on FAMEs using an internal standard method (an example with FAMEs from industrial chemical B). Table S3. ^1^H NMR data of (a) the hydrolysed products and (b) the methyl-esterified products from fatty acid zinc salts. Picture S1: Pictures of white precipitation formed in a clear aqueous solution of aqueous work-up solution in (1) upon increase of alkalinity suggesting the formation of Zn(OH)_2_. The same phenomenon was observed in the aqueous work-up solution in 2.3.1.

## References

[1] European Chemicals Agency (March 2019). Substance identification. https://echa.europa.eu/regulations/reach/substance-identity.

[2] ECHA Manual (2017). *How to Prepare Registration and PPORD Dossiers, v. 4.0*.

[3] ECHA Guidance (2016). *Guidance for Identification and Naming of Substances under REACH and CLP, d.v. 2.0*.

[4] Chen B.-L. (2004). Process for preparing fatty acid zinc salts.

[5] Barman S., Vasudevan S. (2006). Melting of saturated fatty acid zinc soaps. *The Journal of Physical Chemistry B*.

[6] Li M., Zhang J., Huang K., Li S., Jiang J., Xia J. (2014). Mixed calcium and zinc salts of dicarboxylic acids derived from rosin and dipentene: preparation and thermal stabilization for PVC. *RSC Advances*.

[7] Fatty Acids, C14-18 and C16-18-Unsatd., zinc salts, September 2019, https://echa.europa.eu/registration-dossier/-/registered-dossier/15088/1

[8] Fatty Acids, Tallow, Zinc Salts, September 2019, https://echa.europa.eu/registration-dossier/-/registered-dossier/11846/1

[9] European Chemicals Agency, Oleochemicals, September 2019, https://echa.europa.eu/support/substance-identification/sector-specific-support-for-substance-identification/oleochemicals

[10] Series on Testing and Assessment No. 193 (2014). *OECD Guidance for Characterizing Oleochemical Substances for Assessment Purposes*.

[11] Christie W. W., Christie W. W. (1993). Preparation of ester derivatives of fatty acids for chromatographic analysis. *Advances in Lipid Methodology-Two*.

[12] Ichihara K. I., Fukubayashi Y. (2010). Preparation of fatty acid methyl esters for gas-liquid chromatography. *Journal of Lipid Research*.

[13] Morrison W. R., Smith L. M. (1964). Preparation of fatty acid methyl esters and dimethylacetals from lipids with boron fluoride-methanol. *Journal of Lipid Research*.

[14] Metcalfe L. D., Schmitz A. A., Pelka J. R. (1966). Rapid preparation of fatty acid esters from lipids for gas chromatographic analysis. *Analytical Chemistry*.

[15] Food Code, Revised 2017, Korea Ministry of Food and Drug Safety, 2017

[16] Ichihara K. I., Shibahara A., Yamamoto K., Nakayama T. (1996). An improved method for rapid analysis of the fatty acids of glycerolipids. *Lipids*.

[17] AOAC Official Method 996.06 (2002). *Fat (Total, Saturated, and Unsaturated) in Foods, Revised 2001*.

[18] Ichihara K., Yamaguchi C., Nishijima H., Saito K. (2003). Preparation of FAME from sterol esters. *Journal of the American Oil Chemists’ Society*.

[19] Kail B. W., Link D. D., Morreale B. D. (2012). Determination of free fatty acids and triglycerides by gas chromatography using selective esterification reactions. *Journal of Chromatographic Science*.

[20] Knothe G. H. (2014). *^1^H-NMR Spectroscopy of Fatty Acids and their Derivatives*.

[21] Siciliano C., Belsito E., De Marco R., Di Gioia M. L., Leggio A., Liguori A. (2013). Quantitative determination of fatty acid chain composition in pork meat products by high resolution ^1^H NMR spectroscopy. *Food Chemistry*.

[22] Vlahov G. (1999). Application of NMR to the study of olive oils. *Progress in Nuclear Magnetic Resonance Spectroscopy*.

[23] Gottlieb H. E., Kotlyar V., Nudelman A. (1997). NMR chemical shifts of common laboratory solvents as trace impurities. *The Journal of Organic Chemistry*.

[24] Chemical shift, November 2019, https://www.ucl.ac.uk/nmr/NMR_lecture_notes/L2_3_web.pdf

[25] Bastow T. J., Hodge R. M., Hill A. J. (1997). 1H and 13C NMR studies of water and heavy water absorption in poly (vinyl alcohol) hydrogels. *Journal of Membrane Science*.

[26] Yüksel F., Gül Gürek A., Lebrun C., Ahsen V. (2005). Synthesis and solvent effects on the spectroscopic properties of octatosylamido phthalocyanines. *New Journal of Chemistry*.

[27] Mari S. H., Varras P. C., Wahab A.-t. (2019). Solvent-dependent structures of natural products based in the combined use od DFT calculations and ^1^H-NMR chemical shifts. *Molecules*.

[28] Charisiadis P., Kontogianni V., Tsiafoulis C., Tzakos A., Siskos M., Gerothanassis I. (2014). ^1^H-NMR as a structural and analytical tool of intra- and intermolecular hydrogen bonds of phenol-containing natural products and model compounds. *Molecules*.

[29] Fink J. K. (2017). *Additives for High Performance Applications: Chemistry and Applications*.

[30] Reichle R. A., Mccurdy K. G., Hepler L. G. (1975). Zinc hydroxide: solubility product and hydroxy-complex stability constants from 12.5–75°C. *Canadian Journal of Chemistry*.

