# Supplementary material for: Composition Characterization of Fatty Acid Zinc Salts by Chromatographic and NMR Spectroscopic Analyses on Their Fatty Acid Methyl Esters
Source: J Anal Methods Chem. 2019 Dec 23;2019:7594767. doi: 10.1155/2019/7594767 (PMC6942835; doi:10.1155/2019/7594767)
Supplement: Supplementary Materials — The supplementary data consist of the following Tables S1–S3 and Picture S1: Table S1. Retention times and concentrations of 37 FAMEs in the standard solution. Table S2. Calculation of total fatty acids from GC-MS analysis on FAMEs using an internal standard method (an example with FAMEs from industrial chemical B). Table S3. 1H NMR data of (a) the hydrolysed products and (b) the methyl-esterified products from fatty acid zinc salts. Picture S1: Pictures of white precipitation formed in a clear aqueous solution of aqueous work-up solution in (1) upon increase of alkalinity suggesting the formation of Zn(OH)2. The same phenomenon was observed in the aqueous work-up solution in 2.3.1. [file 7594767.f1.pdf]

Journal of Analytical Methods in Chemistry

Composition Characterization of Fatty Acid Zinc Salts by Chromatographic and NMR Spectroscopic Analyses on their Fatty Acid Methyl Esters

Kwang Seo Park, Yun Ju Kim, and Eun Kyung Choe

Regulatory Chemical Analysis Laboratory, Korea Institute of Industrial Technology, Ansan 15588, Republic of Korea.

Correspondence should be addressed to Eun Kyung Choe; ekchoe@kitech.re.kr

Supplementary Materials

The supplementary data consist of the following Tables S1-S3 and Picture S1 :

Table S1. Retention times and concentrations of 37 FAMEs in the standard solution.
Table S2. Calculation of total fatty acids from GC-MS analysis on FAMEs using an internal standard method (an example with FAMEs from industrial chemical B)
Table S3. ^{1}H NMR data of (a) the hydrolysed products and (b) the methyl esterified products from fatty acid zinc salts.

Picture S1: Pictures of white precipitation formed in a clear aqueous solution of aqueous work-up solution in 2.3.3.1 upon increase of alkalinity suggesting the formation of $Zn(OH)_2$. The same phenomenon was observed in the aqueous work-up solution in 2.3.1.

Table S1: Retention times and concentrations of 37 FAMEs in the standard solution.

Peak No.	RT (min)	Substance Name	Carbon : Double Bond	CAS No.	Certified Conc. (%)	U_{CRM} (%)
1	12.21	Methyl butyrate	C4:0	623-42-7	4.00	± 0.021
2	14.30	Methyl hexanoate	C6:0	106-70-7	4.00	± 0.021
3	17.91	Methyl octadecanoate	C8:0	111-11-5	4.00	± 0.021
4	22.84	Methyl decanoate	C10:0	110-42-9	4.00	± 0.021
5	25.51	Methyl undecanoate	C11:0	1731-86-8	2.00	± 0.011
6	28.20	Methyl laurate	C12:0	111-82-0	4.00	± 0.021
7	30.82	Methyl tridecanoate	C13:0	1731-88-0	2.00	± 0.011
8	33.36	Methyl myristate	C14:0	124-10-7	4.00	± 0.021
9	35.39	Methyl myristoleate	C14:1	56219-06-8	2.00	± 0.011
10	35.78	Methyl pentadecanoate	C15:0	7132-64-1	2.00	± 0.011
11	37.76	Methyl *cis*-10-pentadecenoate	C15:1	90176-52-6	2.00	± 0.011
12	38.13	Methyl palmitate	C16:0	112-39-0	6.00	± 0.032
13	39.72	Methyl *cis*-9-hexadecenoate	C16:1	1120-25-8	2.00	± 0.011
14	40.31	Methyl heptadecanoate	C17:0	1731-92-6	2.00	± 0.011
15	41.89	Methyl *cis*-10-heptadecenoate	C17:1	75190-82-8	2.00	± 0.011
16	42.46	Methyl stearate	C18:0	112-61-8	4.00	± 0.021
17	43.39	Methyl *trans*-9-eladate	C18:1n9t	1937-62-8	2.00	± 0.011
18	43.80	Methyl *cis*-9-oleate	C18:1n9c	112-62-9	4.00	± 0.021
19	44.87	Methyl linolelaidate	C18:2n6t	2566-97-4	2.00	± 0.011
20	45.75	Methyl linoleate	C18:2n6c*	112-63-0	2.00	± 0.011
21	46.44	Methyl arachidate	C20:0	1120-28-1	4.00	± 0.021
22	47.20	Methyl γ-linolenate	C18:3n6	16326-32-2	2.00	± 0.011
23	47.66	Methyl *cis*-11-eicosenoate	C20:1n9	2390-09-02	2.00	± 0.011
24	47.97	Methyl linolenate	C18:3n3	301-00-8	2.00	± 0.011
25	48.28	Methyl heneicosanoate	C21:0	6064-90-0	2.00	± 0.011
26	49.52	Methyl *cis*-11,14-eicosadienoate	C20:2	2463-02-07	2.00	± 0.011
27	50.11	Methyl behenate	C22:0	929-77-1	4.00	± 0.021
28	50.87	Methyl *cis*-8,11,14-eicosatrienoate	C20:3n6	21061-10-9	2.00	± 0.011
29	51.27	Methyl *cis*-13-docosenoate	C22:1n9	1120-34-9	2.00	± 0.011
30	51.60	Methyl *cis*-11,14,17-eicosatrienoate	C20:3n3	55682-88-7	2.00	± 0.011
31	51.83	Methyl tricosanate	C23:0	2433-97-8	2.00	± 0.011
32	51.92	Methyl *cis*-5,8,11,14-eicosatetraenoate	C20:4n6	2566-89-4	2.00	± 0.011
33	53.09	Methyl *cis*-13,16-docosadienoate	C22:2	61012-47-3	2.00	± 0.011
34	53.64	Methyl tetracosanoate	C24:0	2442-49-1	4.00	± 0.021
35	54.20	Methyl *cis*-5,8,11,14,17-eicosapentaenoate	C20:5n3	2734-47-6	2.00	± 0.011
36	54.87	Methyl *cis*-15-tetracosenoate	C24:1n9	2733-88-2	2.00	± 0.011
37	59.66	Methyl *cis*-4,7,10,13,16,19-docosahexaenoate	C22:6n3	2566-90-7	2.00	± 0.011

* C18:2n6c : C18 fatty acid with 2 adjacent cis double bonds starting at C6 counting from the methyl group.

Table S2: Calculation of total fatty acids from GC-MS analysis on FAMEs using an internal standard method (an example with FAMEs from industrial chemical B)

Peak No	RT (min)	Carbon : Double Bond	R_{fi} [a]	P_{ti}	W_{FAMEi} [b]	f_{FAi} [c]	FA (%)	Normalized (%)
5	25.5	C11:0	-	8,982,706	-	0.9300	-	Internal Standard
6	28.2	C12:0	1.0821	4,125,829	0.4294	0.9346	2.56	2.62
8	33.4	C14:0	1.1433	374,575	0.0369	0.9421	0.22	0.23
12	38.1	C16:0	1.1524	7,761,990	0.7586	0.9481	4.58	4.69
16	42.5	C18:0	1.1921	4,000,594	0.3780	0.9530	2.29	2.35
17	43.4	C18:1n9t	1.1650	1,260,657	0.1219	0.9527	0.74	0.76
18	43.8	C18:1n9c	1.1982	133,218,285	12.5225	0.9527	75.99	77.84
20	45.8	C18:2n6c	1.1005	18,095,281	1.8519	0.9524	11.23	11.51
			total				97.62	100

[a,b] R_{fi} and W_{FAMEi} were calculated according to the eqn (1) and (2), respectively.
[b] $f_{FA\,i}$: Conversion factor (fatty acid methyl ester to the corresponding fatty acid)

$$W_{FAMEi} = \frac{P_{ti} * W_{tC11:0} \times 1.0067}{P_{tC11:0} \times R_{fi}} \quad \text{.............eqn (S1)}$$

W_{FAMEi} : Content of each fatty acid methyl ester in test solution
P_{ti} : Peak area of each fatty acid methyl ester in test solution
$W_{tC11:0}$: Weight of internal standard (triundecanoin) added to test portion
1.0067 : Conversion of internal standard (triundecanoin) from triglyceride to FAME
$P_{tC11:0}$: Peak area of internal standard (methyl undecanoate) in test solution
R_{fi} : Response factor of each fatty acid methyl ester

$$R_{fi} = \frac{P_{Si} \times W_{C11:0}}{P_{SC11:0} \times W_i} \quad \text{.............eqn (S2)}$$

P_{Si} : Peak area of each fatty acid methyl ester in standard solution
$P_{SC11:0}$: Peak area of internal standard (methyl undecanoate) in standard solution
$W_{C11:0}$: Weight of internal standard (methyl undecanoate) in standard solution (mg)
W_i : Weight of each fatty acid methyl ester in standard solution (mg)

Table S3 : ^{1}H NMR data of (a) the hydrolysed products and (b) the methyl esterified products from fatty acids zinc salts.

(a) Hydrolysed products from fatty acids zinc salts

Peak No.	1.Industrial Chemical A Hydrolyzed (in DMSO-d6)	2.Industrial Chemical B Hydrolysed (in DMSO-d6)	Multiplicity	Assignments
	Chemical shift, ppm (Integration)			
1	0.85 (3.0)	0.85 (3.0)	Triplet (J=8.0Hz)	Protons of terminal methyl
2	1.16-1.36 (22.6)	1.18-1.36 (19.8)	Multiplet	CH_2 of long chain alkyl
3	1.48 (2.0)	1.48 (2.1)	Quintet (J=6.8Hz)	CH_2 of C3
4	1.91-2.04 (2.1)	1.92-2.04 (3.6)	Multiplet	CH_2-Allylic protons
5	2.17 (2.0)	2.17 (2.0)	Triplet (J=7.6Hz)	CH_2 of C2 (α to COOH)
6	2.65-2.76 (0.19)	2.66-2.77 (0.24)	Multiplet	CH_2-bis-allylic protons
7	-	-	-	-
8	5.27-5.38 (1.2)	5.27-5.38 (2.0)	Multiplet	Olefinic protons
9	11.94 (1.0)	11.94 (0.9)	Singlet	Proton of carboxyl acid

(b) Methyl esterified products from fatty acids zinc salts

Peak No.	1.Industrial Chemical A Methyl Esterified (in $CDCl_3$)	2.Industrial Chemical B Methyl Esterified (in DMSO-d6 + $CDCl_3$)	Multiplicity	Assignments
	Chemical shift, ppm (Integration)			
1	0.86 (3.0)	0.85 (3.0)	Triplet (J=8.0Hz)	Protons of terminal methyl
2	1.15-1.36 (22.2)	1.16-1.35 (19.8)	Multiplet	CH_2 of long chain alkyl
3	1.59 (2.0)	1.51 (2.1)	Quintet (J=6.8Hz)	CH_2 of C3
4	1.96-2.05 (2.0)	1.92-2.03 (3.6)	Multiplet	CH_2-Allylic protons
5	2.28 (2.0)	2.26 (2.0)	Triplet (J=7.6Hz)	CH_2 of C2 (α to $COOCH_3$)
6	2.56-2.87 (0.17)	2.66-2.76 (0.23)	Multiplet	CH_2-bis-allylic protons
7	3.64 (2.8)	3.57 (3.0)	Singlet	Methoxy(OCH_3) protons
8	5.27-5.39 (1.1)	5.25-5.36 (2.0)	Multiplet	Olefinic protons
9	-	-	-	-

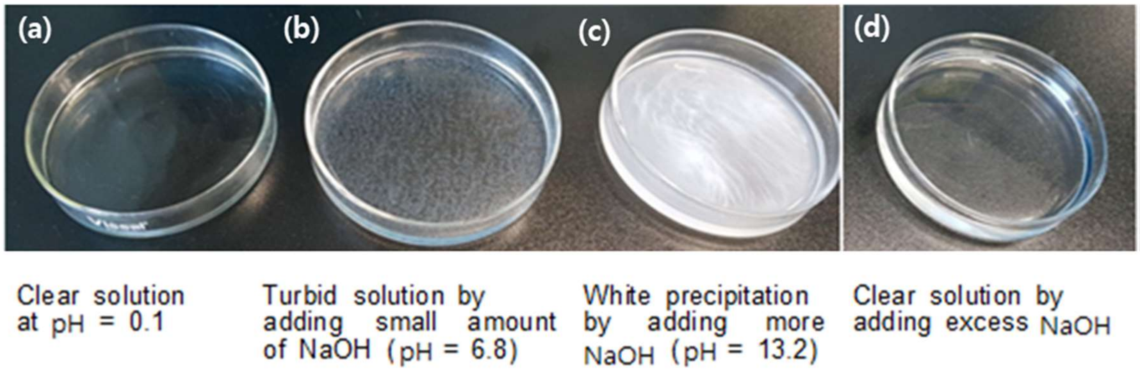

Picture S1: Pictures of white precipitation formed in a clear aqueous solution of aqueous work-up solution in 2.3.3.1 upon increase of alkalinity suggesting the formation of $Zn(OH)_2$. The same phenomenon was observed in the aqueous work-up solutions in 2.3.1.
